# The underlying causes and effects of phytoplankton seasonal turnover on resource use efficiency in freshwater lakes

**DOI:** 10.1002/ece3.7724

**Published:** 2021-06-04

**Authors:** Min Zhang, Xiaoli Shi, Feizhou Chen, Zhen Yang, Yang Yu

**Affiliations:** ^1^ State Key Laboratory of Lake Science and Environment Nanjing Institute of Geography and Limnology Nanjing China

**Keywords:** nitrogen, phosphorus use efficiency, phytoplankton, temporal beta diversity

## Abstract

The extent of intra‐annual turnover in phytoplankton communities is directly associated with the overall diversity. However, our understanding of the underlying causes and effects of intra‐annual turnover remains limited. In this study, we performed a two‐season investigation of the phytoplankton composition in the lakes of the Yangtze River catchment in China in spring and summer 2012, which covered a regional spatial scale. We analyzed the Sørensen pairwise dissimilarity (β_sor_) between the two seasons, their driving factors, and effects on resource use efficiency in phytoplankton. The results showed that the changes in phytoplankton composition from spring to summer were not synchronous among these lakes. The spatial environmental characteristics, temporal changes in environmental variables and the initial phytoplankton composition together explained the variation in β_sor_ for phytoplankton, and their explanatory powers and primary driving variables depended on the phytoplankton taxonomic groups. Among the driving variables, increased nitrogen level and seasonal temperature difference will promote spring–summer community turnover and then improve the phosphorus use efficiency of phytoplankton community. The species diversity of the initial community might increase its stability and slow the spring–summer shift in phytoplankton, especially in cyanobacteria and Chlorophyta. Our study highlights the understanding of the patterns and underlying causes of temporal beta diversity in freshwater phytoplankton communities.

## Graphical abstract

We quantified the contributions of the spatial environmental characteristics, temporal changes in environmental variables, and the initial phytoplankton composition, and deciphered their paths affecting temporal beta diversity. We assessed the difference in response to the three types of variables among taxonomic groups of phytoplankton. We found that increased nitrogen level and seasonal temperature difference will promote spring–summer community turnover and then improve the phosphorus use efficiency of phytoplankton community.

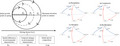

## 1 INTRODUCTION

**FIGURE 1:**
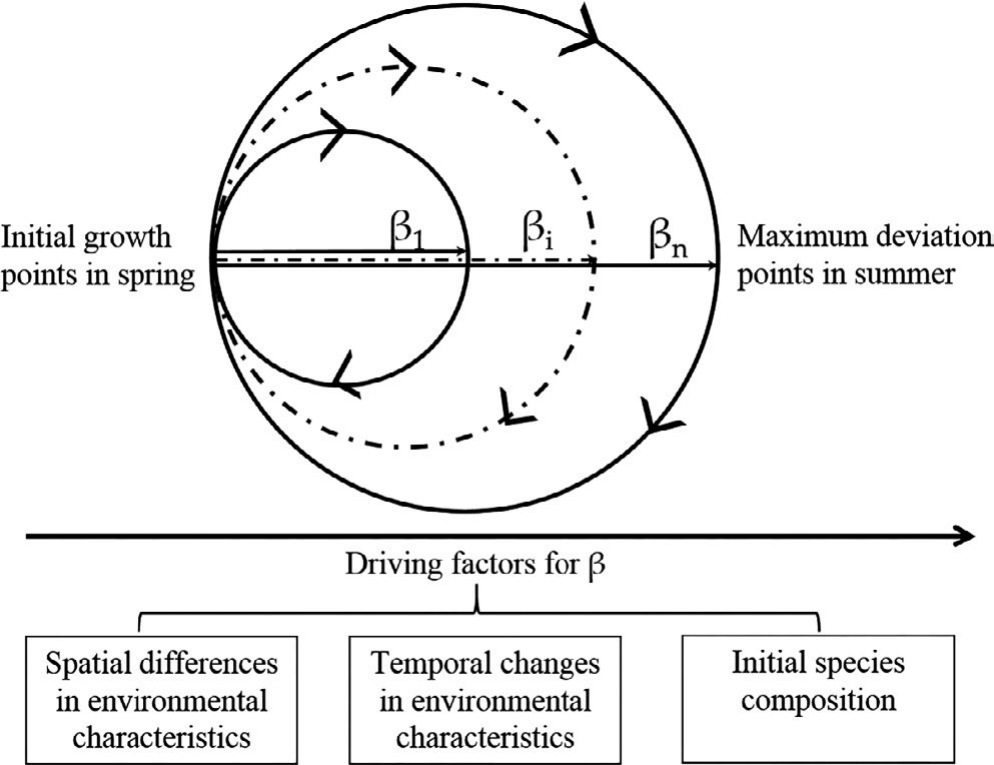
Conceptual basis of the seasonal succession of phytoplankton. The β indicates the temporal beta diversity from the initial growth point in spring to the maximum deviation point in summer. The temporal beta diversity (β*_i_*, the *i*th lake) was different among water bodies (from the first lake, β_1_ to the *n*th lake, β*_n_*) and might be driven by the initial species composition, spatial, and temporal differences in environmental characteristics

The seasonal succession of species communities is associated with the overall diversity at a given site (Bogan & Lytle, 2007; Tonkin et al., 2017). Understanding temporal dynamics is crucial for both basic science and the management of ecosystems (Tonkin et al., 2017). The seasonal succession of phytoplankton is an annually repeated process of community assembly (Sommer et al., 2012). When the growing season begins, the phytoplankton composition starts to shift dramatically and then gradually shifts back after summer following seasonal temperature variations (Figure 1). Therefore, the phytoplankton assemblages in summer may be the most different from those in the nongrowing season (Korhonen et al., 2010). The ranges of the differences among water bodies depend on several ecological, physical, and geographical factors, such as sampling duration, ecosystem size, ecosystem type (lake, stream, or ocean), and latitudinal gradient, and these differences are not uniform across taxonomic groups (Dornelas et al., 2014; Hutchinson, 1961; Korhonen et al., 2010). Although these factors allow for the formulation of specific hypotheses explaining temporal patterns of species distributions at the macroscopic scale (Dornelas et al., 2014), we still do not have a clear understanding of how specific environmental conditions impact the seasonal variability in phytoplankton community composition in freshwater systems and how the seasonal variability further affects the resource use efficiency.

Emerging evidence from natural communities has indicated that beta diversity may vary with interactions among multiple variables (Alves‐de‐Souza et al., 2017; Chalcraft et al., 2004; Chase & Leibold, 2002; Korhonen et al., 2010). In particular, phytoplankton composition is influenced by the complex interplay among many factors that sometimes include temperature, stratification, inorganic nutrients, light availability, zooplankton grazing, and allelopathy (Roelke & Spatharis, 2015). Among the factors that may cause temporal variations in species compositions, environmental heterogeneity is thought to play a major role (Veech & Crist, 2007). Environmental heterogeneity includes spatial differences in environmental characteristics (e.g., eutrophic gradients among lakes) and temporal changes in these environmental characteristics along with temporal beta diversity (e.g., the amplitude of seasonal temperature differences among lakes) (O'Reilly et al., 2015). Previous studies have found that eutrophication reduces the seasonal variation in macroinvertebrate assemblages (Cook et al., 2018), while warming increases the spatial beta diversity of macroinvertebrates, benthic diatoms, macrophytes (Hillebrand et al., 2010) and bacterioplankton (Ren et al., 2017). However, the contributions of these two aspects to temporal beta diversity are unclear. In addition, the initial species composition might also contribute to the variation in temporal beta diversity because diverse communities are known to maintain more temporal stability, and it has often been suggested that they should be more resilient to environmental change (Allan et al., 2011; Shurin, 2007).

For phytoplankton, separating different taxonomic groups might also favor the assessment due to the functional differences among them (Alves‐de‐Souza et al., 2017; Hillebrand et al., 2010), although functional differences among taxonomic groups are smaller than those among functional classification groups (Reynolds et al., 2002). For example, cyanobacteria are frequently found in still or slowly flowing freshwaters and are sensitive to increasing nitrogen and phosphorus levels (Brand, 1996). Diatoms are particularly abundant at the beginning of spring when the water tends to contain plenty of nutrients, and their biological intervention in the movement of silicon is of profound ecological and biogeochemical significance. Therefore, it is possible that there are different underlying processes and many driving factors that determine the shifts in taxonomic groups.

Many previous studies have presented their findings about seasonal succession in different waterbodies or time scales. However, given the high degree of seasonal variation inherent in phytoplankton assemblages, examining the effects at a single time point across a spatial metacommunity (i.e., a watershed) or examining the effects at a specific water across a time scale may be insufficient to fully explain the impact on communities due to the lack of time intervals or environmental gradients. Thus, spatiotemporally explicit studies are needed to examine the effects of these variables on the temporal assembly process with equal time intervals, as focusing on their spatial or temporal effects alone may mask important variation in biodiversity (Wojciechowski et al., 2017). The aim of this study was to analyze the temporal beta diversity in different phytoplankton taxonomic groups and their responses to three types of variables: spatial differences in environmental characteristics, temporal changes in environmental variables, and the initial species composition. To decipher the specific underlying drivers in determining temporal beta diversity, we implemented two synchronous field investigations in spring and summer at a regional scale in the lakes located in the Yangtze River catchment, China, which span the obvious gradient variations in the three types of variables. Specifically, we first tested how the temporal beta diversity among the taxonomic groups was related to the spatial environmental characteristics, temporal changes in environmental variables, and the initial community composition. We expected that the contribution of spatial environmental characteristics would be greater than that of other variables to the variability in cyanobacterial and diatom communities. Second, we tested the hypothesis that eutrophication will decrease the spring–summer beta diversity of phytoplankton by homogenizing their habitats in spring and summer. We predicted at least that the spring–summer beta diversity of cyanobacteria would decrease with increasing eutrophication. Third, we tested the hypothesis that the species diversity in spring will slow down the spring–summer shift in phytoplankton.

## 2 METHODS

### 2.1 Study lakes

**FIGURE 2:**
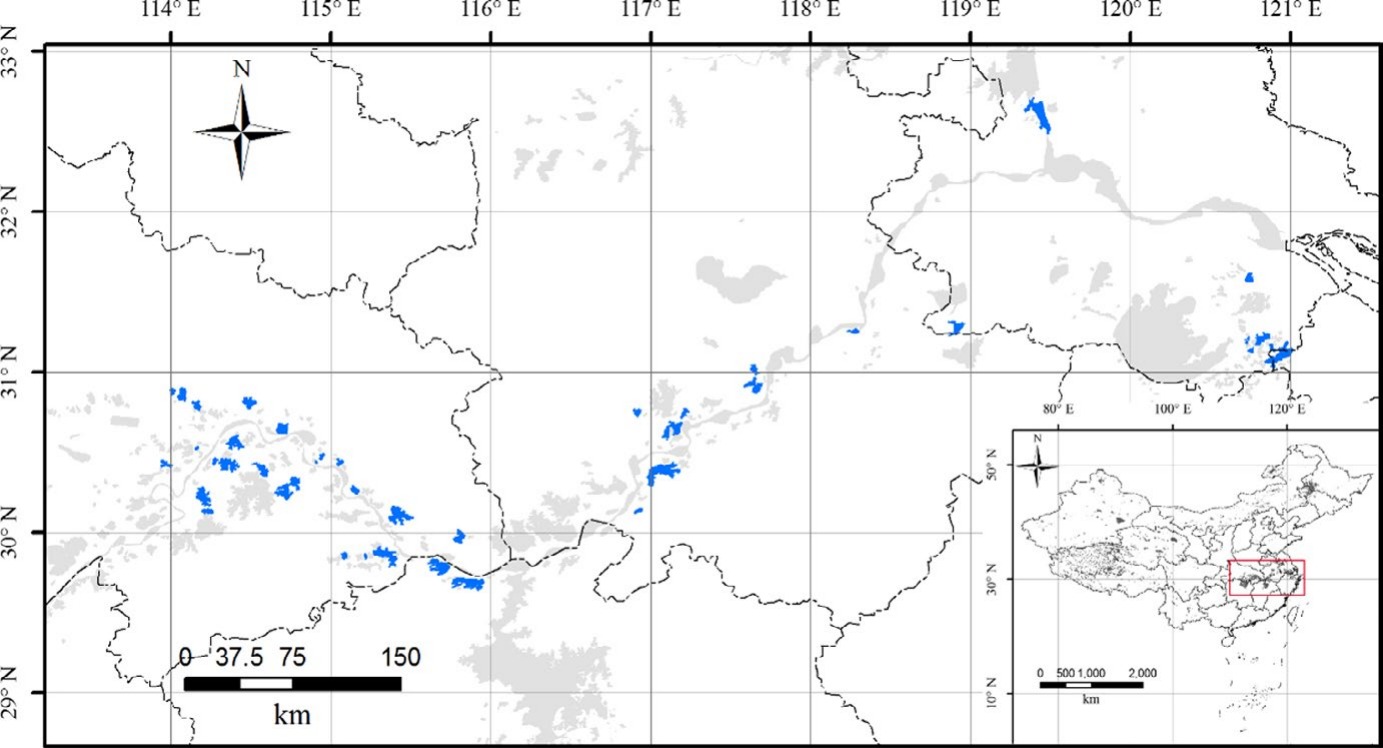
Map of the study lakes in China. The gray polygons indicate all the lakes (area >1 km^2^) in China, and the blue polygons indicate the lakes investigated in this study

Forty‐nine floodplain lakes (all areas >1 km^2^, median/interquartile range of lake area: 16.1/27.8 km^2^), along the Yangtze River in China, from the middle reaches to the lower reaches were investigated in this study (Figure 2). This large spatial scale includes diverse systems with different environmental conditions, which is helpful for the assessment of the effects of environmental variables on the different components of temporal beta diversity. All of these lakes were freshwater lakes in subtropical regions that spanned a trophic gradient from oligotrophic to hypereutrophic due to different human population levels (for details on these lakes, see Table S1 and Zhang et al., 2018).

### 2.2 Sampling and analyses

The two synchronous field investigations were carried out in April and August 2012, representing spring and summer, respectively. Each sampling campaign was performed over a short timeframe (approximately 3 weeks) to obtain a snapshot view of the phytoplankton communities in the selected lakes. In each lake, the sampling duration (the interval between the two sampling campaigns) was approximately 120 days, and three sampling sites were integrated to reduce sampling effect. The synchronous sampling campaign excluded the effect of sampling duration and provided the possibility of analyzing the contributions of different types of factors to temporal beta diversity. At each sampling site, the mixed layer depth was determined from the vertical temperature profiles. Integrated water samples (5 L) were collected by mixing the surface (50 cm below the surface), middle, and bottom (50 cm above the bottom) samples taken with a Uwitec water sampler (Uwitec, Mondsee, Austria).

Vertical profiles of physical and chemical parameters (temperature, pH, dissolved oxygen (DO), and conductivity) were measured at every sampling site to calculate the mean values and determine the depth of the mixed layer using a multiparameter meter (model 6600V2; Yellow Springs Instruments, Yellow Springs, OH, USA). The results showed that all the lakes were shallow and polymictic. Transparency (*SD*) was measured with a Secchi disk. Five liters of water were collected for laboratory analyses according to the method (APHA, 1985; Valderrama, 1981). The total nitrogen (TN), dissolved total nitrogen (DTN), total phosphorus (TP), and dissolved total phosphorus (DTP) concentrations were analyzed using peroxodisulphate oxidation and the spectrophotometric method. Ammonium (${\text{NH}}_{4}^{+}$), nitrate (${\text{NO}}_{3}^{‐}$), nitrite (${\text{NO}}_{2}^{‐}$), and dissolved inorganic phosphorus (${\text{PO}}_{4}^{3‐}$) were measured using a continuous flow analyser (Skalar SA 1,000, Breda, The Netherlands). Chlorophyll *a* was extracted with 90% acetone and measured on a spectrofluorophotometer (Shimadzu RF‐5301PC, Japan) (Yan et al., 2004). The dissolved ions (Cl^−^, ${\text{SO}}_{4}^{2‐}$, K^+^, Na^+^, Ca^2+^, and Mg^2+^) were analyzed using ion chromatography, inductively coupled plasma atomic emission spectroscopy, or inductively coupled plasma mass spectrometry. We calculated the mean light (*I_m_*) value using the following formula (Riley, 1957):

$$I_{m}=I\frac{1‐e^{‐KZ_{m}}}{KZ_{m}}$$

Here, we used the diurnal mean light during the sampling period as the surface light (*I*). We estimated the extinction coefficients (*K*) using the Secchi disk transparency (*Z*_sd_) with the relationship, *K* = 1.54/*Z*_sd_ (Sterner, 1990). Because the temperature at different depth differs from the lake surface temperature by no more than 1℃, the mixing depth (*Z_m_*) was calculated as the water depth at each site. The diurnal mean light during the sampling period was obtained from the closest meteorology stations of the China Meteorological Administration.

### 2.3 Phytoplankton analysis

Integrated 500‐mL phytoplankton samples were collected at each site and fixed with acid Lugol's solution. The identification was performed at the species or genus level using the most recent literature (Hu & Wei, 2006). Counts were conducted in random fields (more than 30 fields) in sedimentation chambers (30 ml) using an inverted microscope following the criteria of Utermöhl (1958). For the dominant species, at least 100 individuals were counted. For all lakes, we considered the organism as the unit (unicell, colony, or filament) to facilitate the calculation of the biovolume. The cell numbers per colony as well as the organism dimensions, including the maximum linear dimension, were estimated. The biovolume was calculated from the measurements of 30 organisms of each species at each site according to Hillebrand et al. (1999). The biomass was determined as the algal volume for each lake and converted to a fresh weight assuming a specific gravity of 1 g/cm^3^. The species richness was the total number of species recorded during the counts, which was standardized to the count size.

### 2.4 Data analysis

In the study, we divided all the analysis into four steps as the list in the following data analysis, which also were descripted in details as an outline graph for the procedures (Figure S1).

#### 2.4.1 Visualizing community differences and calculating the temporal beta diversity

To diminish the effect of rare species, we removed the species that contributed <1% of the total community biomass in each sample and occurred in <3 lakes. Nonmetric multidimensional scaling (nMDS) was used to visualize the phytoplankton community differences between spring and summer. The correlation networks of species in spring and summer were visualized with the R package “igraph” (Csardi & Nepusz, 2006). We calculated Sørensen pairwise dissimilarity (β_sor_) in each lake between spring and summer using the R package “betapart” (Baselga & Orme, 2012). The temporal beta diversity on the abundance data (log‐transformed) from each lake was calculated with the data on the mean biomass value in each species from three sampling sites. Then, the biomass matrix without rare species was transformed into presence–absence data matrix, which was used to calculate the temporal beta diversity on presence–absence data. We also divided the phytoplankton into different taxonomic groups to calculate their beta diversity indices. The indices of cyanobacteria, Chlorophyta, and Bacillariophyta, which contained the most species of the phytoplankton community (accounting for 28%, 36%, and 19%, respectively), were used to perform the following analysis. The indices of the other groups, such as Dinophyta, Chrysophyta, Cryptophyta, and Euglenophyta, were not included in the following analysis due to their large random error derived from relatively rare species (less than 4 species). The values of all these beta diversity indices were compared with Tukey's test.

#### 2.4.2 Analyzing the underlying causes for the variation in temporal beta diversity

To identify the primary reasons for the variation in the temporal beta diversity, the relationships between the temporal beta diversity and the potential explanatory variables were analyzed with generalized additive models (GAMs). We classified all measured variables into three types: spatial environmental characteristics, the temporal changes in environmental variables, and the initial community characteristics in spring. To decrease the degrees of freedom to below the number of sampled lakes, we first performed PCA to reduce the dimensions of the spatial environmental variables presenting as their mean values (electronic conductivity, TN, DTN, TP, DTP, Cl^−^, ${\text{SO}}_{4}^{2‐}$, K^+^, Na^+^, Ca^2+^, Mg^2+^, ${\text{PO}}_{4}^{3‐}$, ${\text{NH}}_{4}^{+}$, NOx including ${\text{NO}}_{2}^{‐}$, and ${\text{NO}}_{3}^{‐}$) and the temporal changes in environmental variables presenting as the coefficients of variation (CVs) of these environmental variables between spring and summer before GAMs analysis. The CVs were calculated with two mean values of these variables in spring and summer. PCA was performed with the R package “psych.”

For the mean values of the environmental variables, we got three principal components: the first principal component PCA_Ion_, including electronic conductivity and the concentrations of dissolved ions (Cl^−^, ${\text{SO}}_{4}^{2‐}$, K^+^, Na^+^, Ca^2+^, and Mg^2+^); the second principal component PCA_P_, including TP, DTP, and ${\text{PO}}_{4}^{3‐}$; and the third principal component PCA_N_, including TN, DTN, ${\text{NH}}_{4}^{+}$, and NOx (Table S2). These principal components were used as explanatory variables for temporal beta diversity. The remaining variables (temperature, DO, pH, and *I_m_*) were used as explanatory variables without the PCA step due to their independency in a correlation analysis. The temperature, DO, and *I_m_* were log‐transformed [log_10_ (*x* + 0.0001)] before analysis to reduce the distributional skew.

For the CVs in environmental variables, we also got three principal components: the first principal component PCA_Ioncv_, including the CVs of electronic conductivity and the concentrations of dissolved ions (Cl^−^, ${\text{SO}}_{4}^{2‐}$, K^+^, Na^+^, Ca^2+^, and Mg^2+^); the second principal component PCA_Pcv_, including TP.cv, DTP.cv, and ${\text{PO}}_{4}^{3‐}$.cv; and the third principal component PCA_Ncv_, including TN.cv, DTN.cv, ${\text{NH}}_{4}^{+}$.cv, and NOx.cv (Table S3). The CVs of temperature, DO, pH, and *I_m_* were used as explanatory variables without the PCA step due to their independency in a correlation analysis.

For the initial phytoplankton community characteristics in spring, community composition (i.e., sample scores on the first two axes of nMDS, MDS1 and MDS2) and species richness were chosen as explanatory variables.

After obtaining the variables, the relationships between the beta diversity components in the total phytoplankton and taxonomic groups and these explanatory variables were analyzed with GAMs with Gaussian errors and restricted maximum likelihood (REML) with the smoothness selection method using the “mgcv” package in R (Wood, 2007). The GAMs fit the smoothing functions of the independent variables and hence permit nonlinear relationships between the dependent and independent parameters. The estimated degrees of freedom (e.*df*) indicate the degree of nonlinearity of the GAMs: e.*df* values close to 1 imply linear relationships, e.*df* values >1 imply progressively higher‐order relationships, and e.*df* values close to zero indicate that the estimated smoothings for a specific independent variable have virtually been removed from the model. When the smoothing of an independent variable was nonsignificant or their e.*df* values were close to 0, we refitted the model without the independent variables to verify that the smoothing of the parameters remaining in the models had not changed and that the model deviances had not substantially increased. The proportions of the variance in the temporal beta diversities were obtained from deviance explained by the GAMs, and the results were presented in diagrams. The significant explanatory variables derived from the models for three types of variables were used for testing their effects on nutrient use efficiency.

#### 2.4.3 Examining the relationships between temporal beta diversity and explanatory variables

We also explored the relationships between the three types of variables and temporal beta diversity in total phytoplankton and taxonomic groups using partial least squares path modeling (PLS‐PM) in the R package “plspm”. This method is known as the partial least squares approach to structural equation models (*SEM*) and allows the estimation of complex cause–effect relationship models with latent variables (Sanchez, 2013). Four latent variables were used: spatial differences of environment characteristics (TN, DTN, ${\text{NH}}_{4}^{+}$, NOx, and water temperature), temporal changes in environmental characteristics from spring to summer (the CVs of TP and water temperature), phytoplankton composition in spring (richness and the first two axes of nMDS), and temporal beta diversity (β_sor_ in phytoplankton and taxonomic groups). The observed variables were selected based on their loadings for latent variables. Most of them were more than 0.7 (Figure S2). We ran PLS‐PM using 1,000 bootstraps to validate the estimates of the path coefficients and coefficients of determination (Wang et al., 2016). The performance of the models was evaluated using the goodness of fit statistic (Sanchez, 2013).

#### 2.4.4 Testing the effects of primary explanatory variables on nutrient use efficiency

We first calculated the nitrogen and phosphorus use efficiency of phytoplankton community as the ratios of the phytoplankton and taxonomic group biomass to TN or TP using all data for 49 lakes. Then, the relationships between these ratios (log‐transformed) and the primary explanatory variables derived from the results of GAMs were fitting with linear and quantile regressions. All data analyses were performed using the code written with R for Windows 3.3.2 (R‐Core‐Team, 2014).

## 3 RESULTS

### 3.1 Spring–summer beta diversity

**FIGURE 3:**
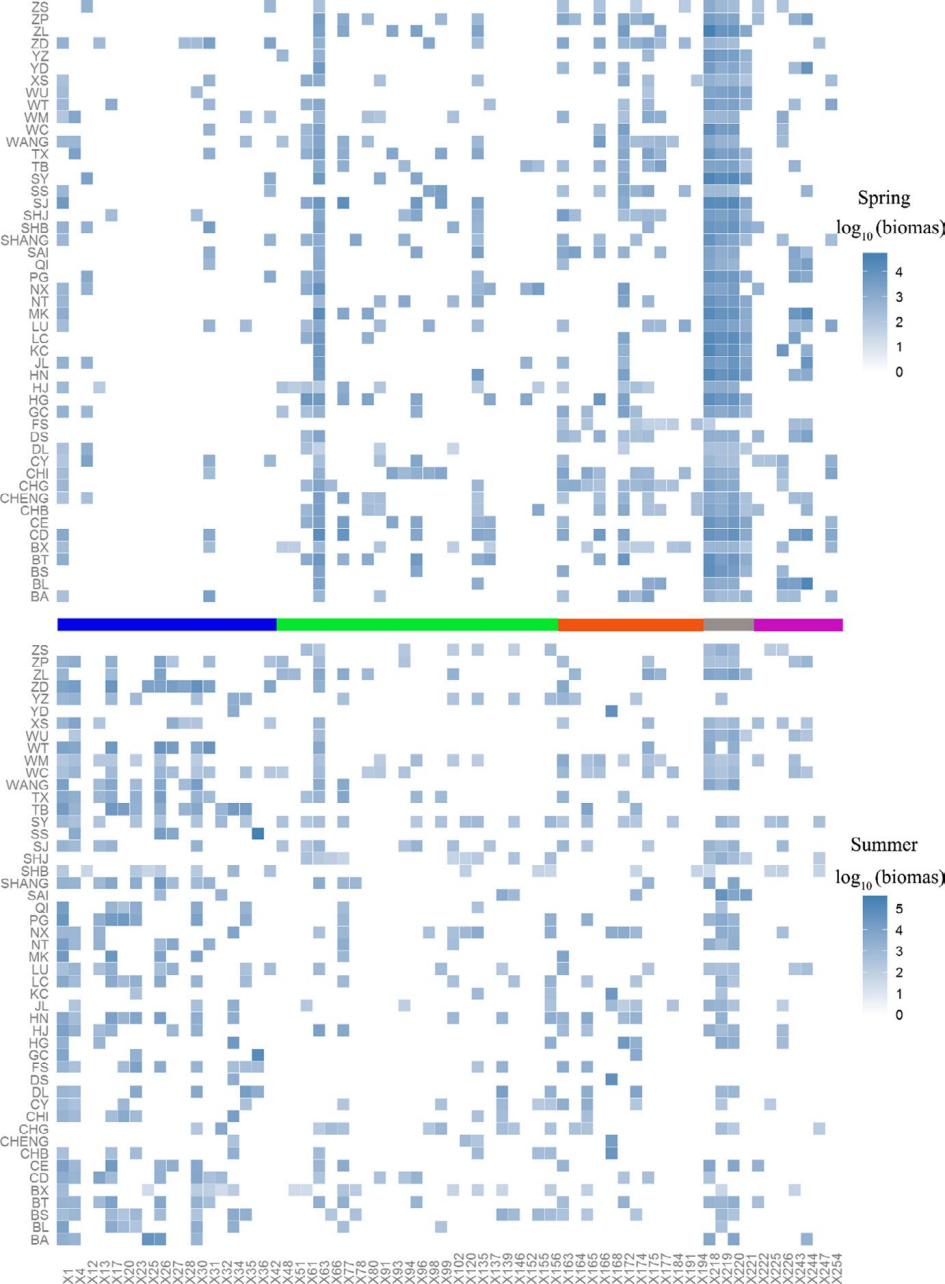
Heat maps of the species composition in spring and summer among the lakes. The steel‐blue color gradient represents the log‐transformed biomass in the two seasons. The color bar between the two panels was used for differentiating the species in the phytoplankton taxa groups. Blue: cyanobacteria; green: Chlorophyta; orange: Bacillariophyta; gray: Cryptophyta; purple: others. The species names that correspond to the labels on the *x*‐axis are shown in Table S4

**FIGURE 4:**
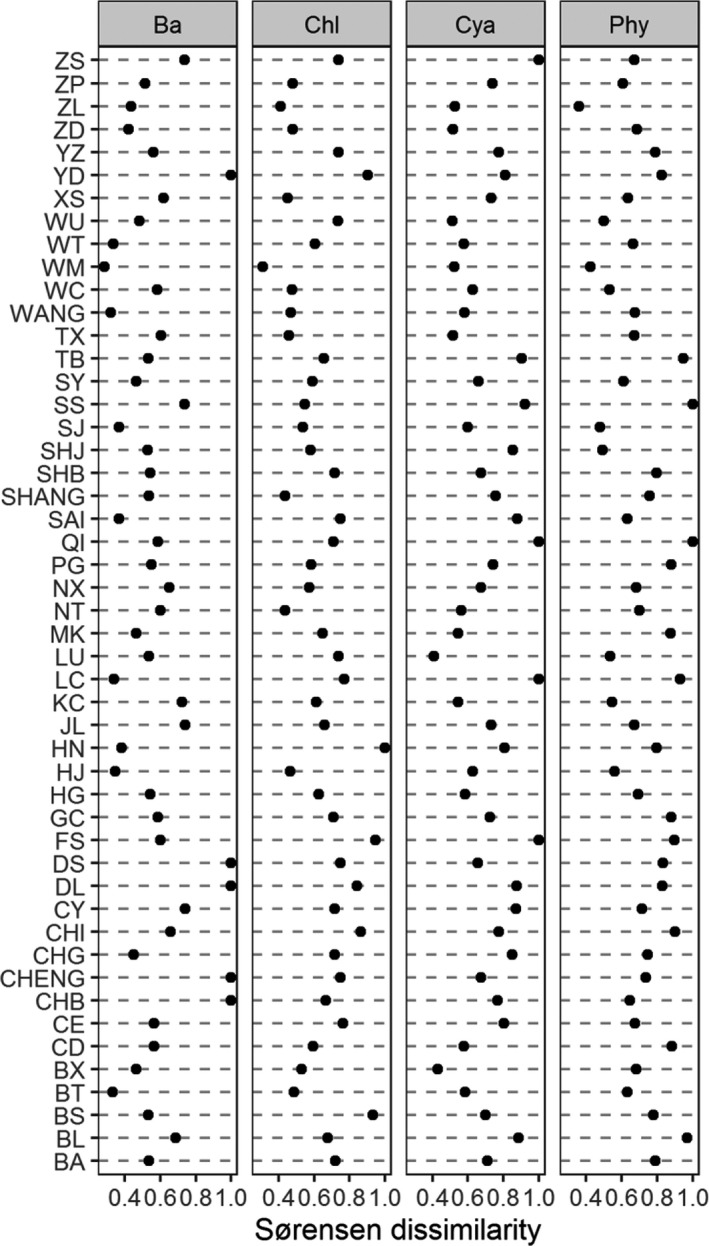
The spring–summer Sørensen pairwise dissimilarity of phytoplankton taxonomic groups (Phy, total phytoplankton; Cya, Cyanobacteria; Chl, Chlorophyta; Ba, Bacillariophyta) based on abundance data in each lake

The phytoplankton composition dynamically changed from spring to summer according to the species distributions (Figure 3). The number of common cyanobacteria species in these lakes increased and those in the other taxonomic groups decreased during the process (Figure 3 and Table S4). The β_sor_ values based on abundance and binary data were high and reached 0.72 and 0.71, respectively (Figures 4 and S3). The β_sor_ values based on abundance and binary data showed significant differences among taxonomic groups, cyanobacteria > Chlorophyta > Bacillariophyta (HSD test, *p* < .05, Figure S4). The network correlation among these species in summer was higher than that in spring. The compositions in spring were similar among lakes and the distribution of lakes was concentrated, but the distribution in summer was dispersed in the nMDS ordination plot (Figure S5).

### 3.2 The underlying causes of the variation in temporal beta diversity

The variation in β_sor_ for the total phytoplankton among the lakes was explained by the three types of variables. The CVs of the environmental variables had the highest explanatory power (28.8%), followed by the mean values of the environmental variables (21.8%) and the initial phytoplankton composition (13.0%). The PCA_N_, the CV of temperature, and MDS2 were the primary factors affecting the variation in β_sor_ among the lakes (Table 1).

**TABLE 1:** Summary of GAMs relating temporal beta diversity of phytoplankton taxonomic groups (abundance data) to environmental variables, CV of environment and phytoplankton composition in spring (full model) and to them separately

Group	Variables	Full			Environment			CV of environment			Phytoplankton in spring		
		e.*df*	*F*	Deviance explained	e.*df*	*F*	Deviance explained	e.*df*	*F*	Deviance explained	e.*df*	*F*	Deviance explained
Phytoplankton	PCA_N_	0.000	0.000^ns^	42.10%	0.775	1.146*	21.80%						
	Tem.cv	0.838	1.726*					1.672	3.936**	28.80%			
	MDS2	0.863	2.106**								1.235	2.256**	13%
Cyanobacteria	PCA_Ion_	2.050	7.130***	61.20%	1.924	5.288***	50.80%						
	pH	0.340	0.712^ns^		0.858	2.009**							
	Tem	0.713	0.826*										
	pH.cv	1.344	1.688*					0.935	4.827***				
	Im.cv	0.849	1.876*										
	richness	0.097	0.036^ns^								0.812	1.442*	21.20%
	MDS1	0.000	0.000^ns^								0.851	1.910*	
Chlorophyta	PCAp	1.811	7.461***	72.30%	1.349	1.327˙	46.40%						
	PCA_N_	0.891	2.714**		0.792	1.266*							
	Tem	1.780	4.352**		1.561	1.855*							
	pH	1.493	3.075**		1.420	2.510**							
	PCA_Pcv_	0.887	2.623**										
	Tem.cv	0.000	0.000^ns^					1.535	2.400*	22.20%			
	pH.cv	0.000	0.000^ns^					0.796	1.302*				
	Im.cv	1.654	2.608*										
	richness	0.862	2.089**								0.896	2.866**	16.80%
	MDS2	1.267	1.443*										
Bacillariophyta	PCA_N_	0.713	0.829˙	52.80%	0.849	1.880*	44.70%						
	PCA_Ion_	0.925	4.091***		0.936	4.904***							
	pH	0.905	3.157**		0.875	2.337**							
	Tem.cv	0.805	1.379*					0.650	0.619˙	39.20%			
	PCA_Pcv_	0.341	0.135^ns^					1.686	3.594**				
	PCA_Ioncv_	0.755	0.500^ns^					1.383	2.197*				
	richness	0.000	0.000^ns^								0.79	1.250*	8.77%

Note

For all variables in the models, the estimated degrees of freedom (e.*df*) and *F* values with corresponding significance levels (***p < .001, **p < .01, *p < .05, ˙*p* < .1, ns *p* > .05) are shown. Model performance is indicated by the deviance explained (dev. expl.) of the respective models.

Abbreviations: PCA_N_, the principal component scores of nitrogen nutrients including the mean values of total nitrogen, dissolved total nitrogen, nitrite, nitrate and ammonia; PCA_P_, the principal components of phosphorus nutrients including the mean values of total phosphorus, dissolved total phosphorus, phosphate; pH, the pH mean values in spring and summer; PCA_Ion_, the principal components of the electronic conductivity and the concentration of dissolved ions; Tem, water temperature; PCA_Ncv_, the principal component scores of the CV of nitrogen nutrients including the CV of total nitrogen, dissolved total nitrogen, nitrite/nitrate, and ammonia; PCA_Pcv_, the principal components of the CV of phosphorus nutrients including the CV of total phosphorus, dissolved total phosphorus, phosphate; PCA_Ioncv_, the principal components of the CV of electronic conductivity and the concentration of dissolved ions; pH.cv, the CV of pH; Tem.cv, the CV of water temperature; Im.cv, the CV of underwater mean light; richness, MDS1 and MDS2, the species richness and the first two axes of nMDS.

The variation in β_sor_ for cyanobacteria among the lakes was mainly explained by the mean values of the environmental variables (50.8%) and the explanatory power for the CVs of the environmental variables (27.2%) and the initial species composition (21.2) was similar (Table 1). The ionic concentrations, pH, the CVs of pH and temperature, richness, and MDS1 were the primary explaining variables for the variation in β_sor_ of cyanobacteria (Table 1).

The variation in β_sor_ for Chlorophyta among the lakes was mainly explained by the mean values of environmental variables (46.4%), followed by the CVs of the environmental variables (22.2%) and the initial phytoplankton composition (16.8%). The phosphorus and nitrogen level, temperature, pH, the CVs of pH and temperature, and richness were the primary explanatory variables for the β_sor_ variation of Chlorophyta (Table 1).

The variations in β_sor_ for Bacillariophyta among the lakes were mainly explained by the mean values of environmental variables (44.7%), followed by the CVs of the environmental variables (39.2%) and the phytoplankton composition (8.8%). The contributions of the phytoplankton composition to the variations were lower than those for cyanobacteria and Chlorophyta. The nitrogen and ionic concentrations level, pH, CVs of temperature, phosphorus and ionic concentrations, and richness were the primary explanatory variables for the β_sor_ variation in Bacillariophyta (Table 1).

**FIGURE 5:**
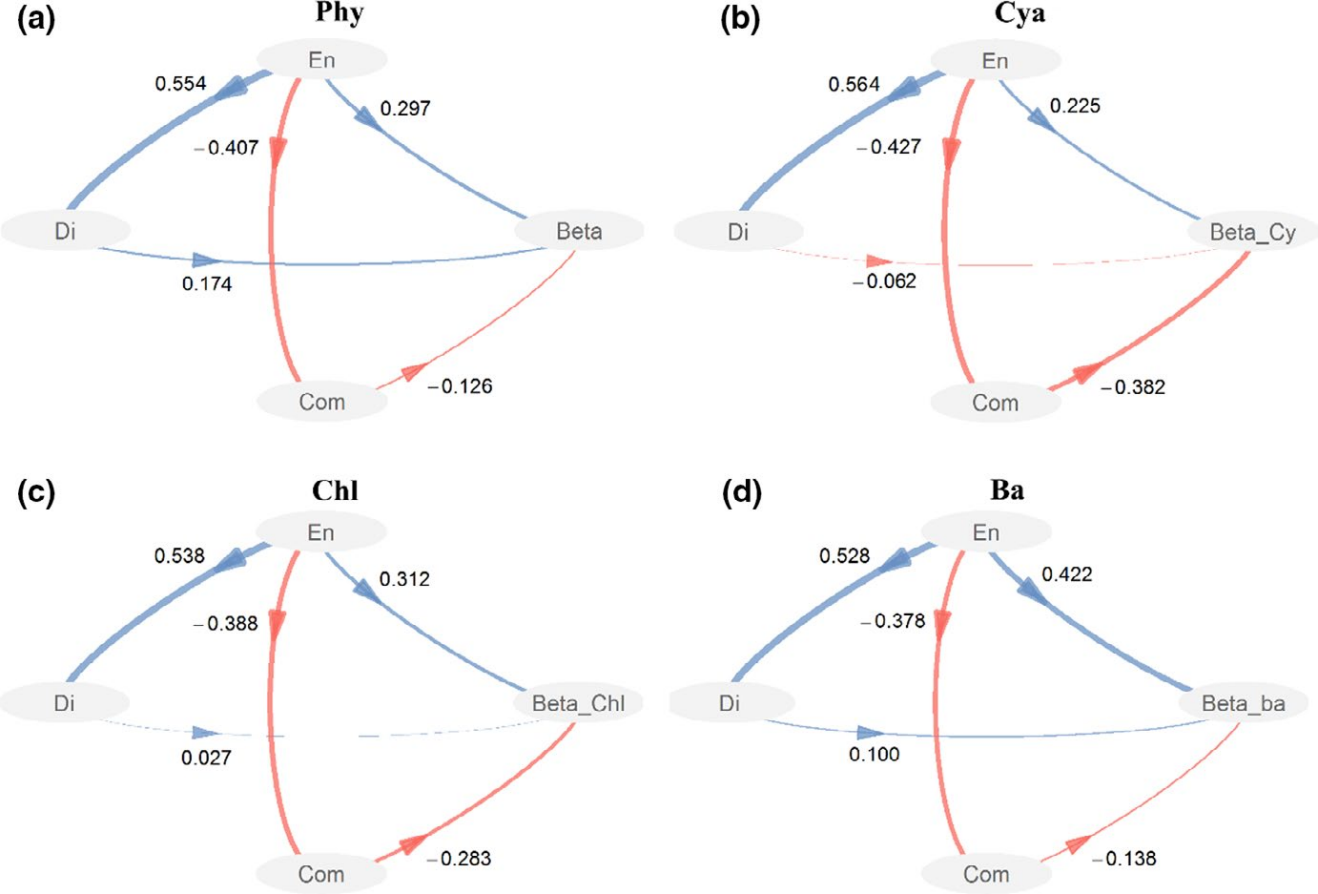
The effects of spatial differences of environment characteristics (En), temporal changes in environmental characteristics from spring to summer (Di), and phytoplankton composition in spring (Com) on Sørensen dissimilarity (based on the abundance data) for phytoplankton (a, Phy) and taxonomic groups (b, cyanboacteria: Cya; c, Chlorophyta: Chl; and d, Bacillariophyta: Ba), which were explored with partial least squares path model. For spatial differences of environment characteristics, TN, DTN, ${\text{NH}}_{4}^{+}$, NOx, and water temperature were selected as the observed variables. For temporal changes in environmental characteristics from spring to summer, the observed variables included the CVs of TN and water temperature. For phytoplankton composition in spring, species richness and the first axis of nMDS were used as observed variables. The path coefficients were calculated after 1,000 bootstraps

According to the results of PLS‐PMs, the mean values and CVs of the environmental variables had positive effects on temporal beta diversity in phytoplankton and taxonomic groups, while phytoplankton composition had negative effects (Figure 5). The negative effect of phytoplankton composition on β_sor_ in Bacillariophyta was lower than those in cyanobacteria and Chlorophyta, and the positive effect of the mean values of environmental variables on cyanobacteria was higher than those in Chlorophyta and Bacillariophyta. These results were similar to those of GAMs, which suggest that there was an obvious difference in driving forces for temporal beta diversity among taxonomic groups.

**FIGURE 6:**
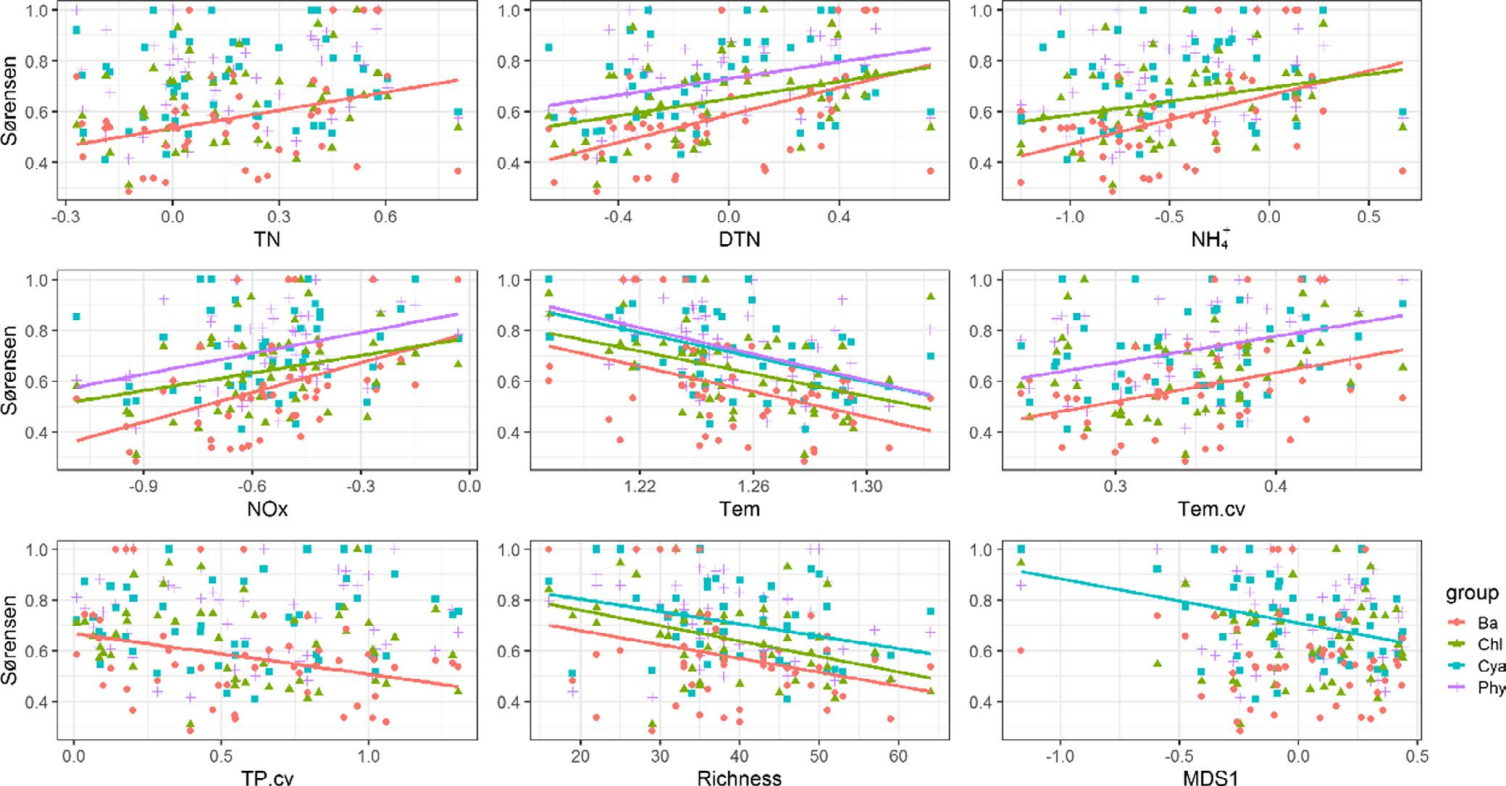
Changes in the Sørensen dissimilarity index based on the abundance data along total nitrogen (TN), dissolved total nitrogen (DTN), ammonium (${\text{NH}}_{4}^{+}$), nitrate and nitrite (NOx), water temperature (Tem), the CV of water temperature (Tem.cv), the CV of total phosphorus (TP.cv), richness, and the first axis of nMDS gradients. The points in different shapes and colors indicate phytoplankton taxonomic groups (Phy, total phytoplankton; Cya, Cyanobacteria; Chl, Chlorophyta; Ba, Bacillariophyta). The solid line indicates the significant linear fit (*p* < .05)

To synthesize the results of GAMs and PLS‐PMs, the nitrogen level, the CV of temperature, and phytoplankton composition in spring were the primary factors affecting the variation in β_sor_ among the lakes. The β_sor_ for the total phytoplankton and taxonomic groups increased significantly with increasing nitrogen level and the CV of temperature and decreased with increasing richness and the first two axes of nMDS gradient (Figure 6, Figures S6 and S7).

### 3.3 Nitrogen and phosphorus use efficiency of phytoplankton community

**FIGURE 7:**
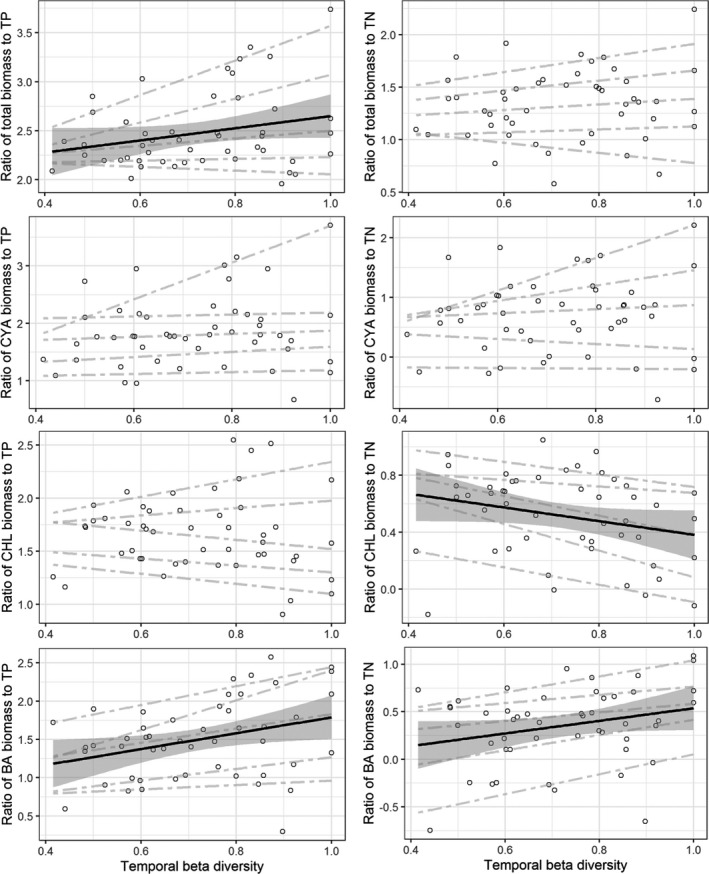
Variation in phosphorus and nitrogen use efficiency of total phytoplankton (log‐transformed) and taxonomic group community (CYA, cyanobacteria; CHL, Chlorophyta; BA, Bacillariophyta) along the temporal beta diversity. The solid line indicates the significant linear fit (*p* < .05). The gray area is approximately 95% confidence intervals on the fitted function. The dash lines were the quantile regression lines (10%, 25%, 50%, 75%, and 90%)

With the increase in temporal beta diversity, the range of nitrogen and phosphorus use efficiency of phytoplankton community obviously increased. The trend of the nitrogen use efficiency increased or decreased along with the temporal beta diversity. And the phosphorus use efficiency showed significantly increasing trend (Figure 7, *p* < .05). In the taxonomic groups, the phosphorus use efficiency of Bacillariophyta increased significantly with the increasing temporal beta diversity (*p* < .05), and there were no obvious trends in those of cyanobacteria and Chlorophyta. The nitrogen use efficiency of Bacillariophyta also showed significantly increasing trend, while that of Chlorophyta decreased significantly (*p* < .05).

## 4 DISCUSSION

### 4.1 Variations in phytoplankton from spring to summer

Among these lakes, the extents of the variations in the phytoplankton composition were remarkably different. In some lakes, the phytoplankton composition almost completely shifted, and in other lakes, the composition only partially shifted. We found that the lakes in spring were concentrated within a relatively small area in the nMDS ordination plot, which indicated that the phytoplankton compositions among the lakes were similar in spring. However, in summer, the distribution of the lakes was dispersed, and the phytoplankton composition was different from that in spring. This result suggests that the phytoplankton composition in the lakes obviously changed from spring to summer, and the amplitudes of these changes were not synchronous, which magnified the difference in the phytoplankton composition among these lakes in summer. These results clearly indicate that the patterns can differ in lakes with different habitat characteristics, which was also found for rivers (Soininen & Eloranta, 2004) and oceans (Defriez et al., 2016). In addition, composition synchrony is dependent on the synchrony of a specific population in a community (Anderson et al., 2019; Defriez & Reuman, 2017). Therefore, the weak spatial synchrony in composition suggests that the variation in some species was not synchronous from spring to summer. These species occurred or disappeared during the shift in most of the lakes, and there were obvious differences among the lakes. For example, *Dolichospermum* sp. and *Raphidiopsis* sp. were rarely found in these lakes in spring, but occurred in most of the eutrophic lakes in summer.

In all the lakes, most species in spring also occurred in summer, and only a few species disappeared or appeared during the succession from spring to summer. However, β_sor_ for each specific lake exhibited a broad range (0.4–1) and its mean value was high (over 0.7). We found that most species were replaced during the process, and a few species only changed their abundance in each specific lake. This might be the reason for high Sørensen dissimilarity in these lakes. Our result is consistent with the results of a previous study (Angeler, 2013; Wojciechowski et al., 2017). These findings suggest that the spring–summer shifts in the phytoplankton community were determined mainly by changing species identities or abundances, not species losses and gains.

### 4.2 Drivers for spring–summer phytoplankton beta diversity

The mechanisms of seasonal succession in phytoplankton communities are quite well studied in both fresh and marine waters (Gasiunaite et al., 2005; Gilabert, 2001; Levasseur et al., 1984; Sommer, 1989). Although the patterns of seasonality within individual bodies of water can be quite regular, comparisons between water bodies often leave the impression of chaos (Harris, 1986). In this study, the phytoplankton assemblages in spring and summer presented large differences in composition, and the differentiation in the temporal beta diversity among the investigated lakes was obvious even though the phytoplankton experienced similar growth durations. Three types of variables, including the environmental characteristics of the lakes, temporal changes in these characteristics, and the initial phytoplankton composition, together explained the variation in the spring–summer beta diversity of phytoplankton. These results emphasize the importance of considering the interaction of multiple environmental factors on the temporal beta diversity of phytoplankton communities, which is consistent with the findings of a previous study (Alves‐de‐Souza et al., 2017).

Among the variables concerning environmental characteristics, the nitrogen level was the primary factor that positively affected the variation in β_sor_ among the lakes, which indicates that eutrophication will increase the spring–summer differentiation in the phytoplankton assemblage and result in relatively dramatic seasonal variation. Some studies have determined the effect of nutrient additions on zooplankton (Gianuca et al., 2017) and macroinvertebrate (Cook et al., 2018) beta diversity. Our findings confirmed the effect in phytoplankton. However, our result was not consistent with the findings for macroinvertebrates, which showed that eutrophication drove sharp reductions in temporal beta diversity (Cook et al., 2018). Differences in body size and life cycle may affect the time required for a response to occur, which may explain the differences (Soininen et al., 2018). In the study, we further determined that nitrogen was the main nutrient that increased the spring–summer shift in phytoplankton. Generally, TN shows obviously decreasing trend from spring to summer. The magnitude of seasonal decrease shows positive correlation with TN concentration (Finlay et al., 2013, and our unpublished data), and the nitrogen composition also changed with increasing TN concentration, especially the ${\text{NH}}_{4}^{+}$ fraction increased significantly (our unpublished data). However, the CV of TN concentration between spring and summer was not the primary factor driving the shift in phytoplankton, which indicated that it might be not the nitrogen loss, but the variation in nitrogen fractions with increasing TN concentration affecting the shift.

Among the variables concerning the temporal changes in the environmental characteristics, the CV of temperature was the primary factor explaining the variation in the β_sor_ with positive correlations. The differentiation in the CV of temperature was mainly attributed to the local climate along the Yangtze River. These results suggest that the spring–summer beta diversity measures were relatively high in the lakes with relatively high seasonal (spring–summer) temperature difference.

Among the variables concerning the phytoplankton community, richness and MDS1 affected the variation in β_sor_ among the lakes. The β_sor_ values decreased significantly with an increase in the community variables, which was positively related to richness and species diversity. These results indicate that species diversity might increase the community stability and slow the spring–summer shift in phytoplankton, which is consistent with previous results for communities of phytoplankton (Ptacnik et al., 2008) and higher organisms, such as macroalgae and zooplankton (White et al., 2006; Shurin, 2007).

### 4.3 Differences in driving factors among taxonomic groups

In this study, we found divergent beta diversity responses in the taxonomic groups to the three types of variables. Compared with the effects observed for Bacillariophyta, the initial community composition had high effects on the variation in β_sor_ for cyanobacteria and Chlorophyta. This indicates that the possible cushion from increasing species diversity and stability had more effect on the spring–summer shifts in cyanobacteria and Chlorophyta than on the shift in Bacillariophyta. The differences in explanatory power of the variables for beta diversity among the taxonomic groups might be due to the species responses to environment or species interactions within communities. Because of their small cell size and short lift cycle, phytoplankton species have evolved unique life‐history strategies to exploit seasonally generated niches during the process of responding to seasonal oscillations in environmental conditions (Beche et al., 2006; Bonada & Resh, 2013), which exert a controlling influence on community composition and diversity (Tonkin et al., 2017). The rapid and divergent responses among taxonomic groups change the seasonal patterns of beta diversity in phytoplankton, which will first lead to trophic mismatch (Straile et al., 2015) and will be followed by variations in the food web structures, biogeochemical processes, and ecological functions (Sommer et al., 2012; Straile et al., 2015).

Eutrophication, especially nitrogen level, did not significantly contribute to the variations in β_sor_ for cyanobacteria, even though cyanobacteria are characterized by fast growth and are directly related to seasonal blooms in eutrophic waters. The response of the temporal beta diversity of total phytoplankton to eutrophication was mainly reflected by the changes in the compositions of Chlorophyta and Bacillariophyta. This finding indicates that the effect of eutrophication on temporal beta diversity is divergent among taxonomic groups. The seasonal temperature difference affected the variation in β_sor_ for Chlorophyta and Bacillariophyta, which might be attributed to their sensitivity to temperature variation. The dynamics of the species in the two taxonomic groups generally are accompanied by rapidly increasing temperature from spring to summer. The species in Bacillariophyta dominate in spring when temperature was relatively low and shift to green algae with increasing temperature, and finally to cyanobacteria in summer. The increase in light availability due to increasing hours of daylight or the melting of the ice cover, which is associated with temperature, might play more important role in these shallow lakes than turbulence or mixing in deep lakes (Peeters et al., 2007).

### 4.4 Resource use efficiency of phytoplankton community

Generally, biodiversity will decrease the temporal community turnover and improve ecosystem functioning, such as resource use efficiency of community, which have been confirmed in phytoplankton community of freshwater lakes and marine systems (Ptacnik et al., 2008; Striebel et al., 2009). However, the relationships between ecosystem functioning and community turnover are inconsistent based on theoretical and experimental studies. Theory predicts that low community turnover will result in the highest rates of ecosystem functioning due to the fact that the best performing species always dominates under stable conditions, and community turnover helps maintain ecosystem functioning under fluctuating conditions (Norberg et al., 2001). The experimental results in grassland systems showed that functional turnover played an important role in maintaining high levels of biomass production (Allan et al., 2011). In the study, we found that the resource use efficiency of phytoplankton increased with increasing temporal beta diversity, which is consistent with the previous findings (Filstrup et al., 2014). Our results showed that the temporal beta diversity was relatively high, and most species were replaced in the intra‐annual scale, which increase the number of species with different and complementary niches. Therefore, turnover of functionally complementary species might be the main reason for high resource use efficiency. Furthermore, nitrogen level and seasonal change in temperature were primary forces driving the variation in temporal turnover, which suggests that the lakes with high nitrogen level and obvious seasonality will have relatively high phosphorus use efficiency of phytoplankton community, which will improve primary production in these lakes, especially for cyanobacterial abundance (Filstrup et al., 2014). The effect of increasing nitrogen level on phosphorus use efficiency of phytoplankton also implicated that decreasing nitrogen level in eutrophic lakes might be helpful for the mitigation of cyanobacterial blooms by decreasing their phosphorus use efficiency.

## 5 CONCLUSIONS

Understanding the effects of beta diversity on ecosystem structure and function is imperative but is complicated by the variety of spatial and temporal extents of observational data (Cardinale et al., 2012; Socolar et al., 2016). Our study demonstrated the amplitude of the seasonal variation in phytoplankton communities in a regional scale by controlling the variety of temporal extents, which was helpful for improving our understanding of the patterns and underlying causes of temporal beta diversity (Alves‐de‐Souza et al., 2017). We found that environmental characteristics, their temporal changes, and the initial community composition together explained the temporal beta diversity of the phytoplankton community. The explanatory powers were obviously divergent among the taxonomic groups. In detail, although eutrophication resulted in similar phytoplankton compositions among these lakes (Zhang et al., 2018), increased nitrogen level promoted the spring–summer community turnover and then improved the phosphorus use efficiency of phytoplankton community. The species diversity of the initial community might increase community stability and slow the spring–summer shift in phytoplankton. Although we controlled the variety of temporal extents and addressed the primary factors affecting the temporal beta diversity, we still found the process to be very complicated. Therefore, more studies examining intra‐annual beta diversity are needed for a robust understanding of community variation and its effects on the structure of food webs, biogeochemical processes, and ecological functions.

## CONFLICT OF INTEREST

None declared.

## AUTHOR CONTRIBUTION

**Min Zhang:** Conceptualization (equal); Formal analysis (lead); Funding acquisition (equal); Investigation (lead); Writing‐original draft (equal); Writing‐review & editing (equal). **Xiaoli Shi:** Conceptualization (equal); Funding acquisition (equal); Writing‐review & editing (equal). **Feizhou Chen:** Funding acquisition (equal); Investigation (lead); Writing‐review & editing (equal). **Zhen Yang:** Funding acquisition (equal); Investigation (equal); Writing‐review & editing (equal). **Yang Yu:** Investigation (equal); Writing‐review & editing (equal).

## Supporting information

Supplementary Material

## Data Availability

All data in this study are freely available at http://lake.geodata.cn/data/datadetails.html?dataguid=272081069608519. https://doi.org/10.11971/lim.2020.001.db
