# Supplementary material for: The underlying causes and effects of phytoplankton seasonal turnover on resource use efficiency in freshwater lakes
Source: Ecol Evol. 2021 Jun 4;11(13):8897–909. doi: 10.1002/ece3.7724 (PMC8258203; doi:10.1002/ece3.7724)

The underlying causes and effects of phytoplankton seasonal turnover on resource use efficiency in freshwater lakes

Min Zhang^*^, Xiaoli Shi, Feizhou Chen, Zhen Yang, Yang Yu

State Key Laboratory of Lake Science and Environment, Nanjing Institute of Geography and Limnology, CAS, Nanjing 210008, China

Supporting Information: Tables and figures

Table S1 The environmental variables (mean±sd) in spring and summer.

|  | Spring | Summer |
| --- | --- | --- |
| Temperature (^o^C) | 17.984±1.243 | 29.843±2.113 |
| Conductivity (Sm/cm) | 0.360±0.170 | 0.335±0.140 |
| pH | 7.920±0.622 | 7.559±1.411 |
| DO (mg/L) | 10.882±1.932 | 7.915±2.672 |
| I_m_ (μmol photons/m^2^•s^1^) | 321.172±148.636 | 163.017±103.248 |
| TN (mg/L) | 1.772±1.174 | 1.554±0.617 |
| DTN (mg/L) | 1.232±1.058 | 0.829±0.385 |
| NH_4_^+^ (mg/L) | 0.572±0.753 | 0.090±0.281 |
| NO_3_^-^ (mg/L) | 0.311±0.163 | 0.196±0.289 |
| NO_2_^-^ (mg/L) | 0.003±0.002 | 0.009±0.013 |
| TP (mg/L) | 0.078±0.070 | 0.227±0.206 |
| DTP (mg/L) | 0.038±0.071 | 0.121±0.140 |
| PO_4_^3-^ (mg/L) | 0.024±0.063 | 0.089±0.136 |
| Cl^-1^  (mg/L) | 26.780±23.022 | 26.839±24.776 |
| SO_4_^-2^  (mg/L) | 59.492±42.325 | 48.474±40.876 |
| Na^+^ (mg/L) | 21.580±21.252 | 10.705±11.466 |
| K^+^ (mg/L) | 4.479±2.067 | 2.760±1.307 |
| Mg^2+^ (mg/L) | 7.963±3.092 | 4.511±1.312 |
| Ca^2+^ (mg/L) | 34.909±12.777 | 20.454±7.016 |

Table S2 Standardized loadings (pattern matrix) based upon correlation matrix of principal components analysis with the average values of variables from spring and summer

|  | PC1 | PC3 | PC2 | h2 | u2 | com |
| --- | --- | --- | --- | --- | --- | --- |
| Conductivity | 0.87 | 0.13 | 0.30 | 0.86 | 0.135 | 1.3 |
| TN | 0.11 | 0.51 | 0.77 | 0.86 | 0.145 | 1.8 |
| DTN | 0.13 | 0.29 | 0.93 | 0.96 | 0.041 | 1.2 |
| TP | 0.13 | 0.87 | 0.17 | 0.80 | 0.201 | 1.1 |
| NH_4_^+^ | 0.19 | 0.34 | 0.84 | 0.86 | 0.139 | 1.4 |
| DTP | 0.28 | 0.86 | 0.27 | 0.88 | 0.116 | 1.4 |
| PO_4_^3-^ | 0.07 | 0.84 | 0.33 | 0.82 | 0.177 | 1.3 |
| Cl^-^ | 0.83 | 0.39 | 0.10 | 0.85 | 0.149 | 1.4 |
| SO_4_^2-^ | 0.88 | -0.06 | 0.17 | 0.82 | 0.185 | 1.1 |
| Na^+^ | 0.85 | 0.33 | 0.07 | 0.83 | 0.166 | 1.3 |
| K^+^ | 0.72 | 0.42 | 0.02 | 0.69 | 0.309 | 1.6 |
| Mg^2+^ | 0.85 | 0.21 | -0.21 | 0.81 | 0.192 | 1.3 |
| Ca^2+^ | 0.74 | -0.17 | 0.11 | 0.59 | 0.412 | 1.2 |
| NOx | -0.01 | 0.00 | 0.77 | 0.60 | 0.400 | 1.0 |
| SS loadings | 4.90 | 3.18 | 3.15 |  |  |  |
| Proportion Var | 0.35 | 0.23 | 0.23 |  |  |  |
| Cumulative var | 0.35 | 0.58 | 0.80 |  |  |  |
| Proportion Explained | 0.44 | 0.28 | 0.28 |  |  |  |
| Cumulative Proportion | 0.44 | 0.72 | 1.00 |  |  |  |

Table S3 Standardized loadings (pattern matrix) based upon correlation matrix of principal components analysis with the coeffient variation of variables from spring and summer

|  | PC1 | PC2 | PC3 | h2 | u2 | com |
| --- | --- | --- | --- | --- | --- | --- |
| Conductivity.cv | 0.06 | 0.08 | 0.14 | 0.03 | 0.97 | 1.90 |
| TN.cv | -0.07 | -0.01 | 0.73 | 0.54 | 0.46 | 1.00 |
| DTN.cv | 0.00 | -0.35 | 0.72 | 0.63 | 0.37 | 1.40 |
| TP.cv | -0.20 | 0.84 | 0.18 | 0.78 | 0.22 | 1.20 |
| NH_4_^+^.cv | 0.13 | 0.07 | 0.43 | 0.21 | 0.79 | 1.20 |
| DTP.cv | -0.33 | 0.59 | 0.32 | 0.56 | 0.44 | 2.20 |
| PO_4_^3-^.cv | -0.41 | 0.24 | 0.43 | 0.41 | 0.59 | 2.60 |
| Cl^-^.cv | 0.14 | 0.49 | 0.09 | 0.27 | 0.73 | 1.20 |
| SO_4_^2-^.cv | 0.34 | 0.68 | -0.16 | 0.61 | 0.39 | 1.60 |
| Na^+^.cv | 0.63 | 0.33 | -0.18 | 0.55 | 0.45 | 1.70 |
| K^+^.cv | 0.75 | 0.17 | 0.27 | 0.67 | 0.33 | 1.40 |
| Mg^2+^.cv | 0.79 | 0.19 | 0.05 | 0.66 | 0.34 | 1.10 |
| Ca^2+^.cv | 0.82 | -0.35 | 0.17 | 0.82 | 0.18 | 1.50 |
| NOx.cv | 0.13 | 0.60 | -0.09 | 0.39 | 0.61 | 1.10 |
| SS loadings | 2.76 | 2.61 | 1.76 |  |  |  |
| Proportion Var | 0.20 | 0.19 | 0.13 |  |  |  |
| Cumulative var | 0.20 | 0.38 | 0.51 |  |  |  |
| Proportion Explained | 0.39 | 0.37 | 0.25 |  |  |  |
| Cumulative Proportion | 0.39 | 0.75 | 1.00 |  |  |  |

Table S4 Species occurred at least in 3 lakes and contributed >1% of total community biomass.

| ID | Phylum | Species name | ID | Phylum | Species name | |
| --- | --- | --- | --- | --- | --- | --- |
| X1 | Cyanophyta | *Microcystis* sp. Lemmermann | X102 | Chlorophyta | *Oocystis borgei* Snow | |
| X4 | Cyanophyta | *Pseudanabaena* sp. Lauterborn | X120 | Chlorophyta | *Tetrastrum* sp. Chodat | |
| X12 | Cyanophyta | *Aphanocapsa elachista* West & G.S.West | X135 | Chlorophyta | *Pectodictyon* sp. Taft | |
| X13 | Cyanophyta | *Merismopedia tenuissima* Lemmermann | X137 | Chlorophyta | *Actinastrum hantzschii* Lagerheim | |
| X17 | Cyanophyta | *Dolichospermum circinalis* Rabenhorst ex Bornet & Flahault | X139 | Chlorophyta | *Mougeotia parvula* Hassall | |
| X20 | Cyanophyta | *Sphaerospermopsis eucompacta* Li & Watanabe | X146 | Chlorophyta | *Treubaria crassispina* Smith | |
| X23 | Cyanophyta | *Dolichospermum* sp. Ralfs ex Bornet & Flahault | X152 | Chlorophyta | *Ulothrix* sp. Kützing | |
| X25 | Cyanophyta | *Raphidiopsis mediterranea* Skuja | X155 | Chlorophyta | *Planctonema* sp. Schmidle | |
| X26 | Cyanophyta | *Raphidiopsis curvata* Fritsch & Rich | X156 | Chlorophyta | Unknow X |  |
| X27 | Cyanophyta | *Raphidiopsis sinensia* Jao | X163 | Bacillariophyta | *Aulacoseira granulata var.angustissima* Müller | |
| X28 | Cyanophyta | *Spirulina* sp. Turpin ex Gomont | X164 | Bacillariophyta | *Aulacoseira granulata var.angustissima f.spiralis* Hustedt | |
| X30 | Cyanophyta | *Planktothrix agardhii* (Gomont) Anagnostidis & Komárek | X165 | Bacillariophyta | *Aulacoseira granulate* (Ehrenberg) Simonsen | |
| X31 | Cyanophyta | *Aphanizomenon gracile* Lemmermann | X166 | Bacillariophyta | *Aulacoseira distans* (Ehrenberg) Simonsen | |
| X32 | Cyanophyta | *Planktothricoides* sp. Suda & Watanabe | X168 | Bacillariophyta | *Aulacoseira* sp. Thwaites | |
| X34 | Cyanophyta | *Aphanizomenon* sp. Morren ex Bornet & Flahault | X172 | Bacillariophyta | *Cyclotella meneghiniana* Kützing | |
| X35 | Cyanophyta | *Aphanizomenon issatschenkoi* (Usacev) Proshkina-Lavrenko | X174 | Bacillariophyta | *Synedra ulna* (Nitzsch) Ehrenberg | |
| X36 | Cyanophyta | *Raphidiopsis raciborskii* (Woloszynska) Aguilera, Berrendero Gómez, Kastovsky, Echenique & Salerno | X175 | Bacillariophyta | *Synedra acus* Kützing | |
| X42 | Cyanophyta | *Lyngbya* sp. Agardh ex Gomont | X177 | Bacillariophyta | *Synedra berolinensis* Lemmermann | |
| X48 | Chlorophyta | *Desmodesmus quadricaudatus* Turpin | X184 | Bacillariophyta | *Navicula* sp. Bory | |
| X51 | Chlorophyta | *Scenedesmus bijuga* (Turpin) Lagerheim | X191 | Bacillariophyta | *Achnanthes exigua* Grunow | |
| X61 | Chlorophyta | *Chlorella* sp. Beyerinck | X194 | Bacillariophyta | *Nitzschia* sp. Hassall | |
| X63 | Chlorophyta | *Chlamydomonas globose* Snow | X218 | Cryptophyta | *Cryptomonas ovata* Ehrenberg | |
| X66 | Chlorophyta | *Pandorina morum* (O.F.Müller) Bory | X219 | Cryptophyta | *Cryptomonas erosa* Ehrenberg | |
| X77 | Chlorophyta | *Coelastrum microporum* Nägeli | X220 | Cryptophyta | *Cryptomonas rostrate* Skuja | |
| X78 | Chlorophyta | *Coelastrum reticulatum* (P.A.Dangeard) Senn | X221 | Cryptophyta | *Chroomonas acuta* Utermöhl | |
| X80 | Chlorophyta | *Chlorogonium elongatum* (P.A.Dangeard) Francé | X222 | Euglenophyta | *Euglena* sp. Ehrenberg | |
| X91 | Chlorophyta | *Kirchneriella* sp. Schmidle | X225 | Euglenophyta | *Euglena oxyuris* Schmarda | |
| X93 | Chlorophyta | *Pediastrum boryanum* [(Turpin) Meneghini](https://www.algaebase.org/search/species/detail/?species_id=27507) | X226 | Euglenophyta | *Euglena caudate* Hübner | |
| X94 | Chlorophyta | *Stauridium tetras* (Ehrenberg) E.Hegewald | X243 | Dinophyta | *Peridinium pusillum* (Pénard) Lemmermann | |
| X96 | Chlorophyta | *Lacunastrum gracillimum (West & G.S.West) H.McManus* | X244 | Dinophyta | *Peridinium bipes* F. Stein | |
| X98 | Chlorophyta | *Monactinus simplex* [(Meyen) Corda](https://www.algaebase.org/search/species/detail/?species_id=119534) | X247 | Dinophyta | *Glenodinium pulvisculus* (Ehrenberg) F. Stein | |
| X99 | Chlorophyta | *Pediastrum simplex* Meyen | X254 | Chrysophyta | *Dinobryon cylindricum* O. E. Imhof | |

Figure S1 The outline graph for the procedures of all necessary statistical analyses in the study. First, we compared the difference in phytoplankton composition between spring and summer using ordination method (nMDS) and networks analysis, and calculated the temporal beta diversity for each lake. Second, we performed the GAMs to find the underlying cause and significant factors for the variation in temporal beta diversity. Third, the PLS-PM analysis was performed to examine the relationships between temporal beta diversity and the three types of explanatory variables. Finally, we tested the effects of primary explanatory variables on nutrient use efficiency.


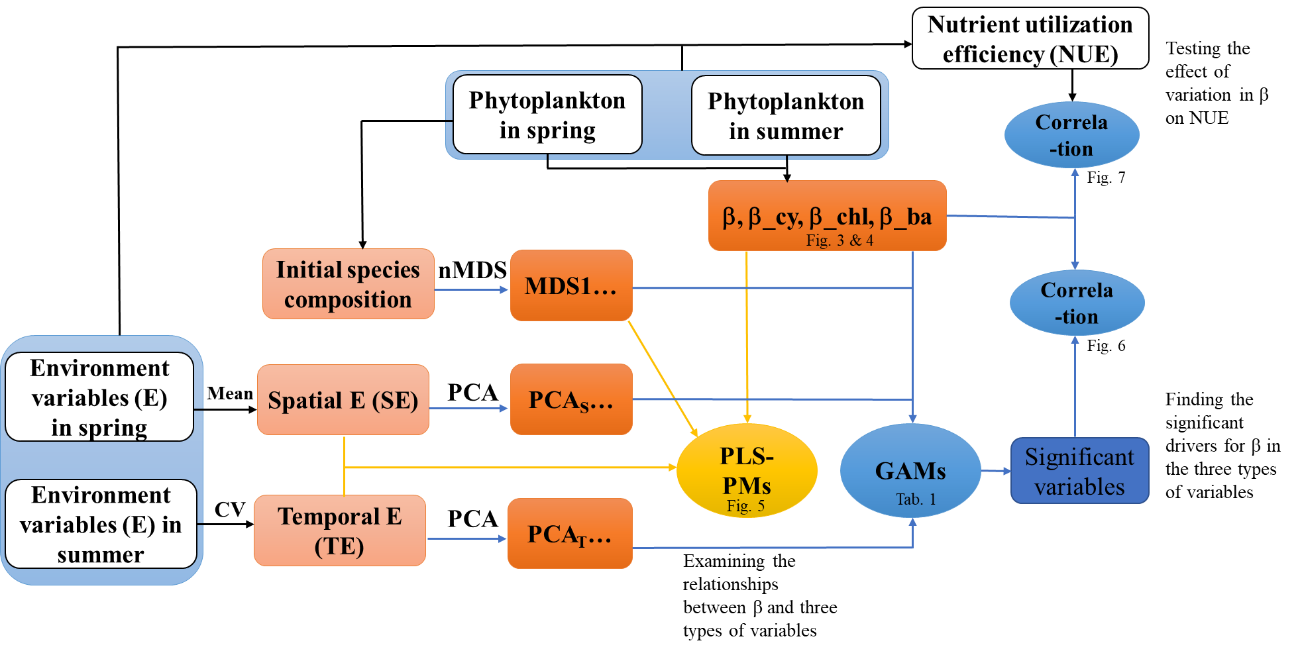


Figure S2 The loadings of observed variables for the latent variables in the partial least squares path models (a, total phytoplankton; b, Cyanobacteria; c, Chlorophyta; d, Bacillariophyta). Most of the loadings were selected with the threshold 0.7. The observed variables were divided into four blocks to establish a set of indicators as latent variables, including spatial differences of environment characteristics (En), temporal changes in environmental characteristics from spring to summer (Di), phytoplankton composition in spring (Com) and temporal beta diversity (Beta, Beta_cy, Beta_chl and Beta_ba in four models, respectively). TN, total nitrogen; DTN, dissolved total nitrogen; NH_4_^+^, ammonium; NOx, nitrate+nitrite; Tem, water temperature; Tem.cv, the CV of water temperature; TP.cv, the CV of total phosphorus; richness, species richness; MDS1, the first axis of nMDS; betadiv, temporal beta diversity.


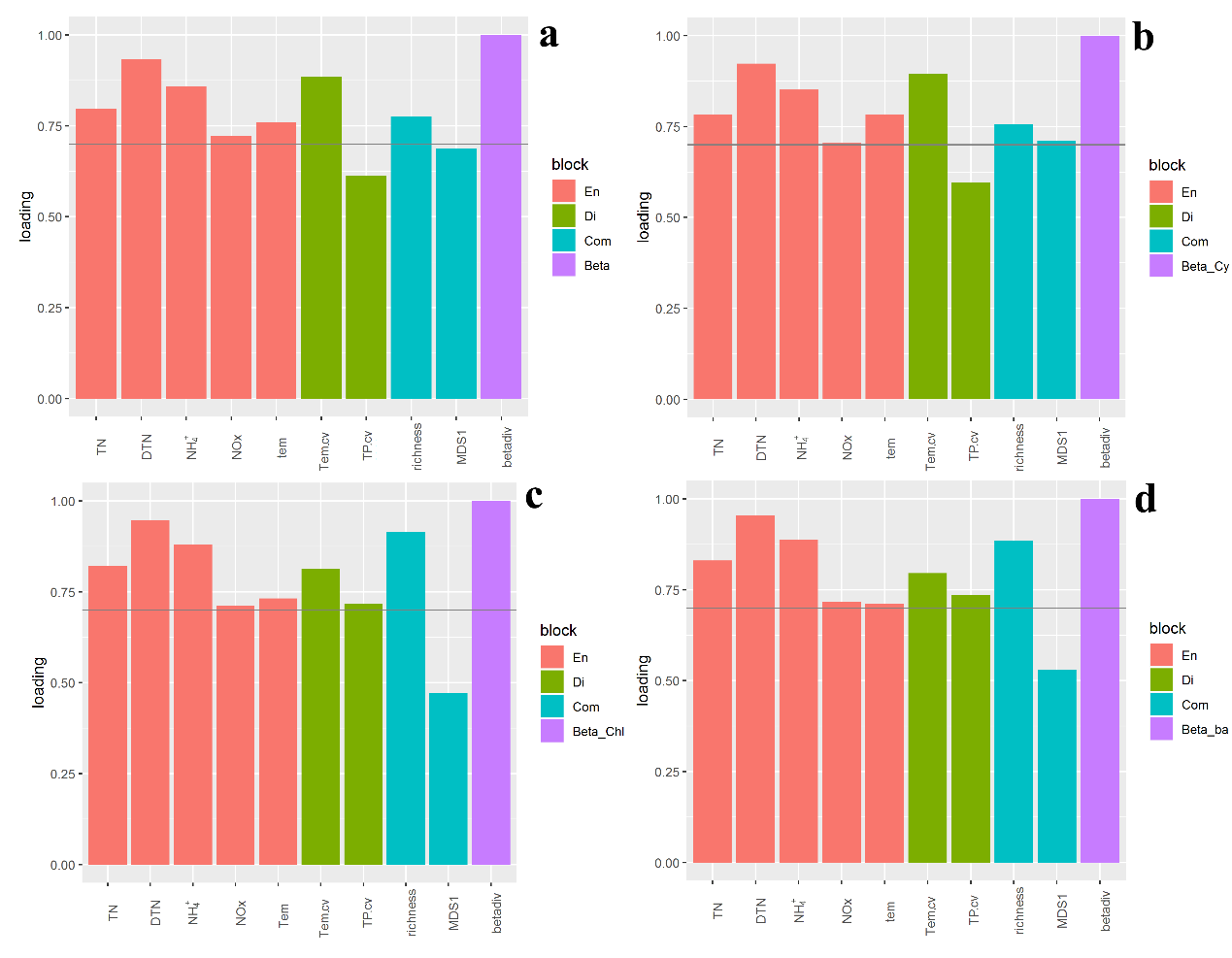


Figure S3 The spring-summer Sørensen pairwise dissimilarity of phytoplankton taxonomic groups (Phy, total phytoplankton; Cya, Cyanobacteria; Chl, Chlorophyta; Ba, Bacillariophyta) based on binary data in each lake.


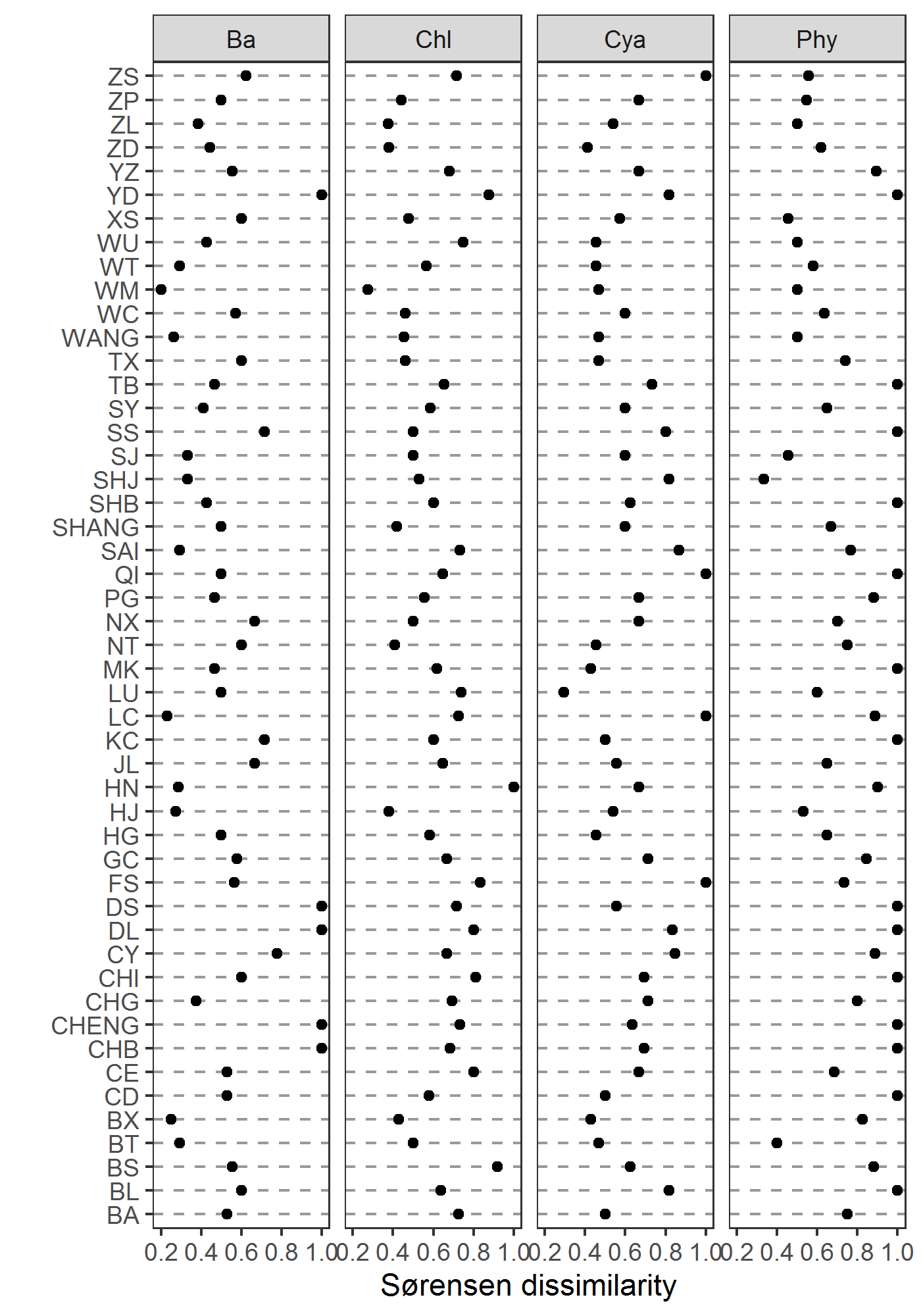


Figure S4 Boxplot of the Sørensen pairwise dissimilarity for phytoplankton taxonomic groups (Phy, total phytoplankton; Cya, Cyanobacteria; Chl, Chlorophyta; Ba, Bacillariophyta) between spring and summer based on the abundance and binary data.


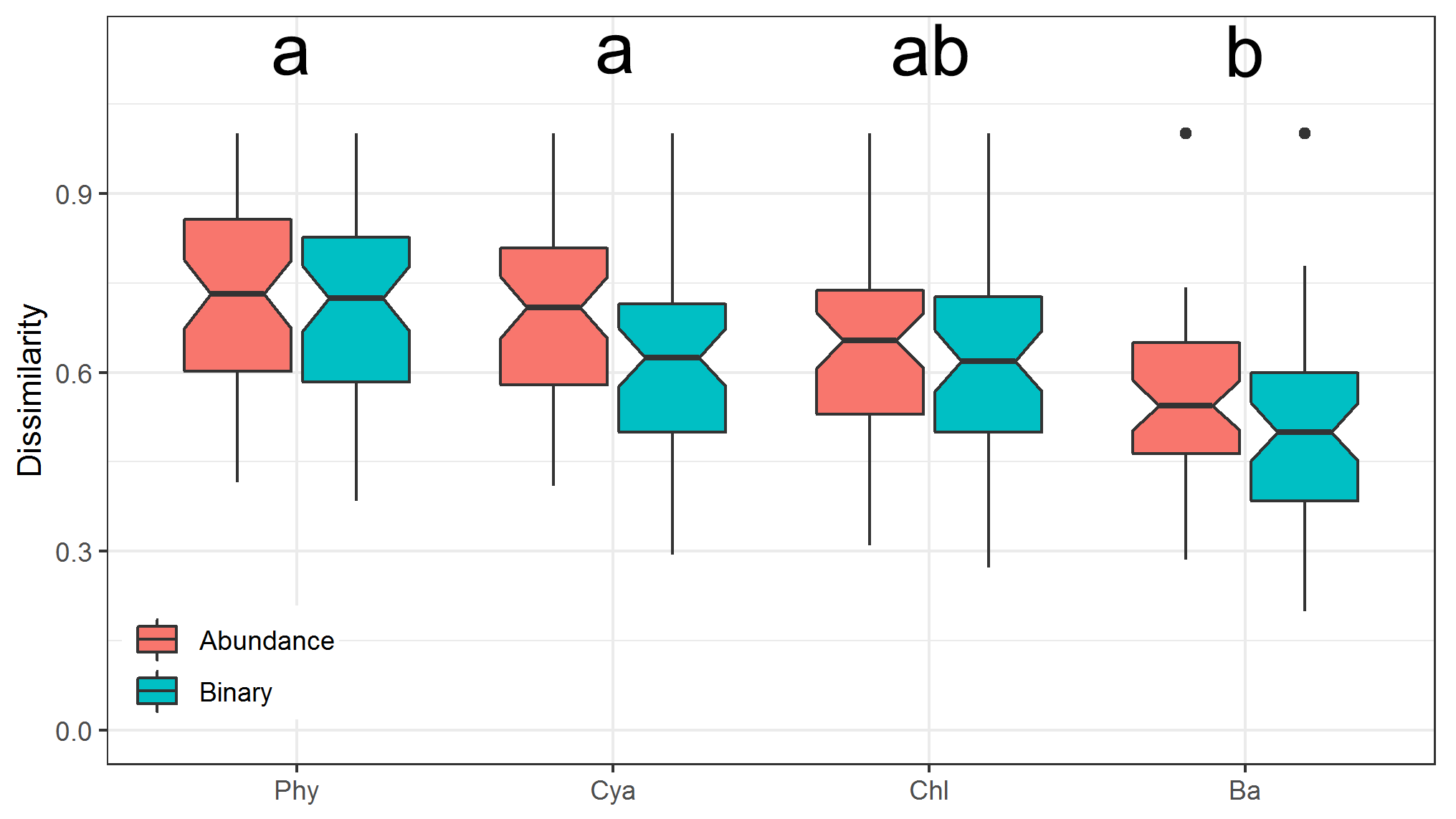


Figure S5 Phytoplankton composition and correlations in spring and summer among the lakes. (a) Correlation networks of species in spring and summer. Every node indicates a species recorded in Figure 3. The node size represents species abundance. Red: species only in spring; green: species only in summer; yellow: co-occurrence species in spring and summer. (b) Nonmetric multidimensional scaling (nMDS) ordination based on the abundance data, where symbols denote the spring (green) and summer (blue) phytoplankton composition in the investigated lakes.


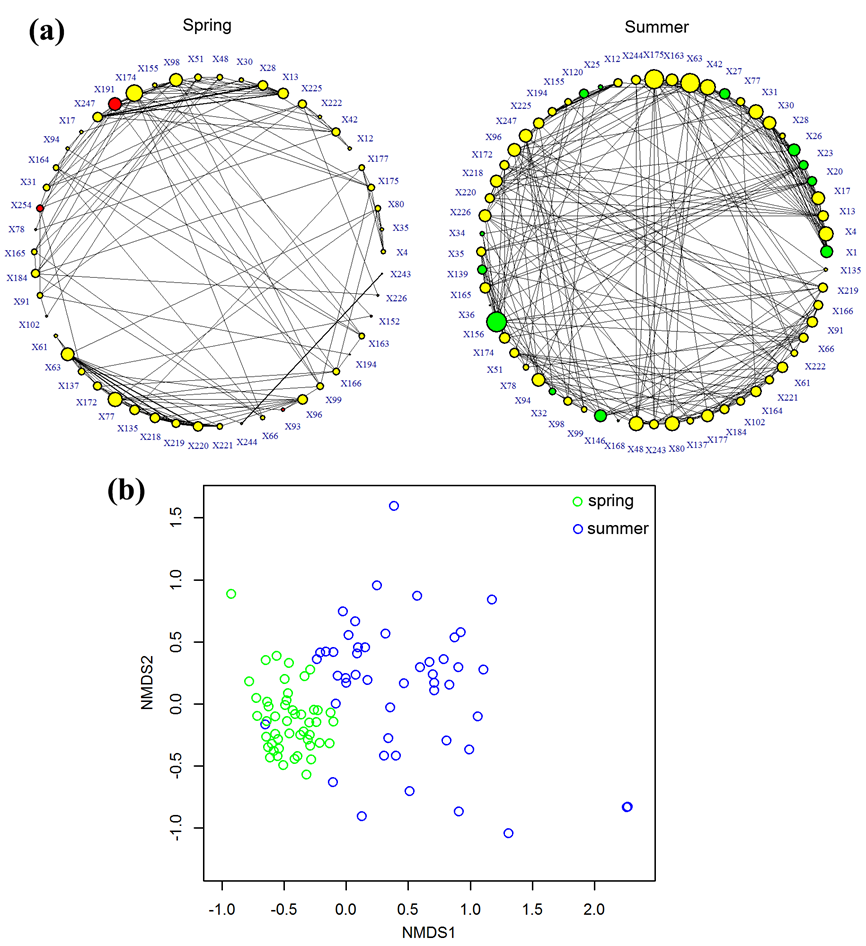


Figure S6 Changes in the Sørensen dissimilarity index based on the abundance data along nitrogen level (PCA_N_), phosphorus level (PCA_p_), dissolved ions level (PCA_Ion_), pH, the CV of pH (pH.cv), the CV of *I_m_* (*I_m_*.cv), nitrate and nitrite (NOx), water temperature (Tem), the CV of water temperature (Tem.cv), the CV of phosphorus level (PCA_Pcv_), the CV of dissolved ions level (PCA_Ioncv_) and the second axis of NMDS gradients (MDS2). The points in different shapes and colors indicate phytoplankton taxonomic groups (Phy, total phytoplankton; Cya, Cyanobacteria; Chl, Chlorophyta; Ba, Bacillariophyta). The solid line indicates the significant linear fit (p<0.05).


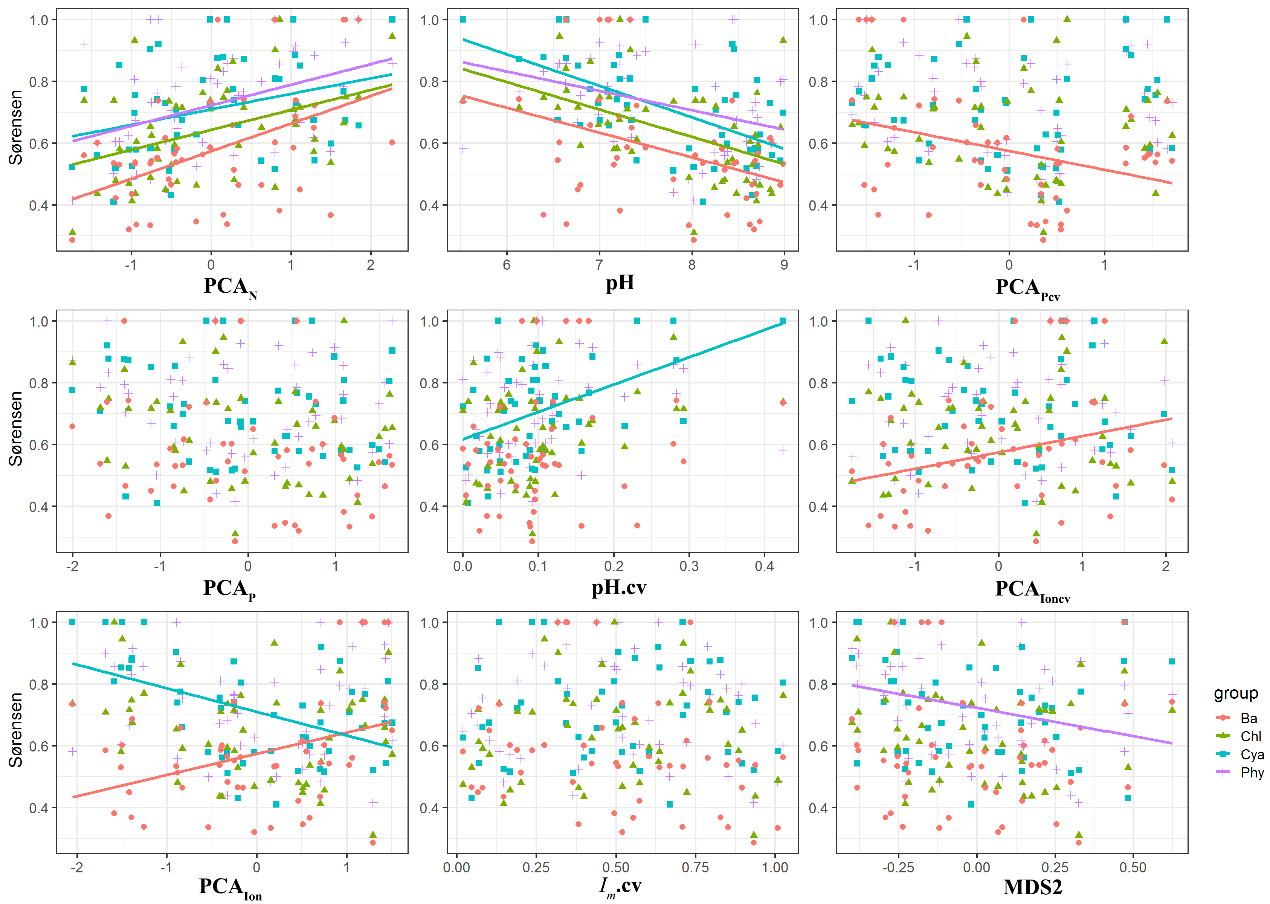


Figure S7 Changes in the Sørensen dissimilarity index based on the binary data along total nitrogen (TN), dissolved total nitrogen (DTN), ammonium (NH_4_^+^), nitrate and nitrite (NOx), water temperature (Tem), the CV of water temperature (Tem.cv), the CV of total phosphorus (TP.cv), richness and the first axis of NMDS gradients. The points in different shapes and colors indicate phytoplankton taxonomic groups (Phy, total phytoplankton; Cya, Cyanobacteria; Chl, Chlorophyta; Ba, Bacillariophyta). The solid line indicates the significant linear fit (p<0.05).


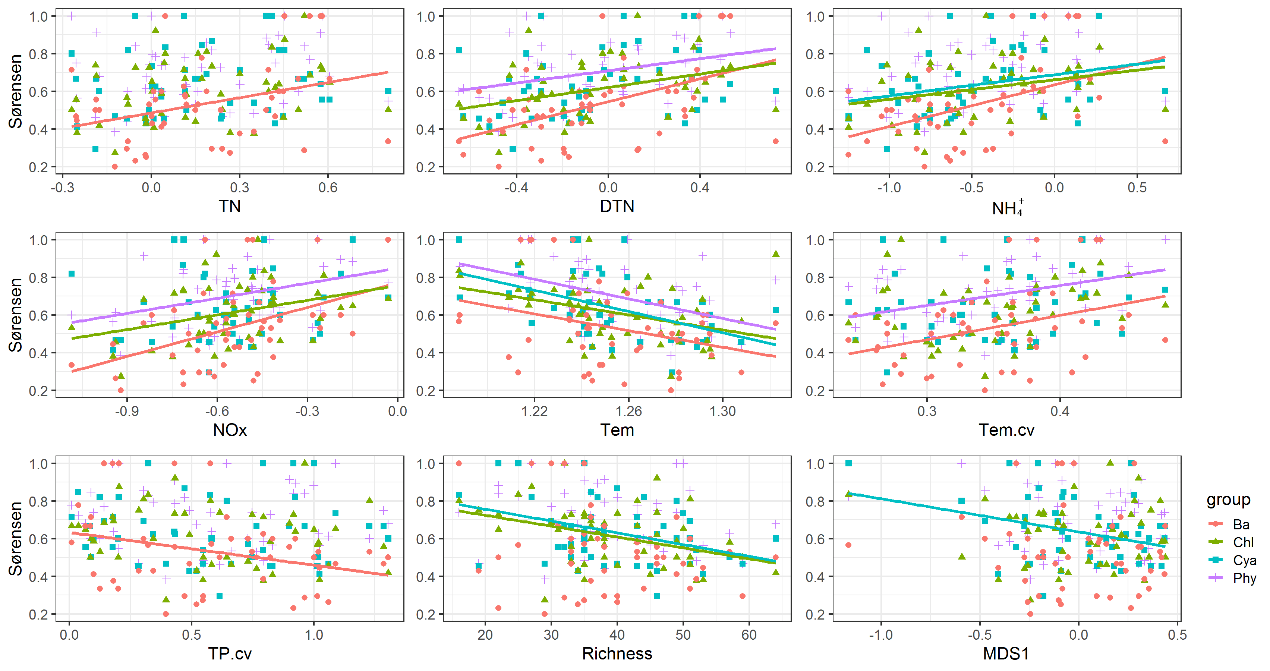

Supplement: Supplementary file 1 — Supplementary Material [file ECE3-11-8897-s001.docx]
